# Parapharyngeal liposarcoma: a case report

**DOI:** 10.1186/1746-1596-8-42

**Published:** 2013-03-07

**Authors:** Hong Li, Xueqin Zhou, Qian Ran, Liuqian Wang

**Affiliations:** 1 Department of Otorhinolaryngology and Head-Neck Surgery, Xinqiao Hospital, PLA, Third Military Medical University, Chongqing, PR, 400037, China; 2 Department of Radiology, Xinqiao Hospital, PLA, Third Military Medical University, Chongqing, PR, 400037, China

## Abstract

**Background:**

Parapharyngeal liposarcoma is a very rare malignant tumor that often causes nonspecific clinical symptoms, such as progressive dysphagia, globus sensation and/or respiratory disturbances. The combination of radiological imaging techniques and histopathological analysis provides information for diagnosis; however, the pathogenesis is still uncertain.

**Case presentation:**

A 30-year-old male patient presented with a pharyngeal cavity mass, which had been present for 2 years. The clinical syndrome included obstructive sleep apnea symptoms (i.e., respiratory disturbances, excessive daytime somnolence, and headache) and difficulty swallowing. The radiological examination (CT) demonstrated that there was a low-density irregular solid lesion on the posterior wall of the oropharynx and laryngopharynx, which descended to the superior mediastinum and extended to the left parapharyngeal space and sternocleidomastoid muscle. The boundaries of the lesion were clear, and the lesion’s density was nonuniform. Several septations inside the lesion were observed. The CT values of the lesion at the epiglottis and the vocal folds were 11 HU and minus 30 HU, respectively. After enhanced scanning, there was no apparent enhancement of the lesion: the surrounding tissue and blood vessels were squeezed and shifted, but the neighboring sclerotin of the cervical vertebrae was not invaded. The mass was removed *via* a transcervical approach, resulting in a complete amelioration of the patient’s symptoms. Interestingly, immunohistochemistry showed that the tumor cells expressed members of the B7 superfamily, including B7-H1, B7-DC and B7-H3. In addition, the expression of TIM-containing molecules, including TIM-3 and TIM-4, was observed.

**Conclusions:**

CT and MRI demonstrated that the mass was a parapharyngeal liposarcoma. Furthermore, carcinoma-associated B7 and TIM-containing molecules were observed in the tissue, indicating that these molecules are most likely active in the pathogenesis of this disease.

**Virtual Slides:**

The virtual slide(s) for this article can be found here: http://www.diagnosticpathology.diagnomx.eu/vs/1907794973876202

## Background

Parapharyngeal liposarcoma is a very rare malignant tumor that often causes nonspecific clinical symptoms, including progressive dysphagia, globus sensation and/or respiratory disturbances [1,2]. These tumors usually grow to a large size before being discovered. The combination of radiological imaging techniques (CT and MRI) and histopathological analysis can provide adequate information for diagnosis. Surgery is the best treatment [3]. Nevertheless, the pathogenesis of this cancer is still uncertain.

The co-inhibitory molecules of the B7 superfamily, including B7-H1 (PD-L1 or CD274), B7-DC (PD-L2 or CD273), B7-H3 (CD276), and B7-H4 (B7x or B7S1), have been demonstrated to actively participate in the regulation of T-cell activation [4,5]. However, it has been reported that many types of carcinomas express these molecules, and the states of these molecules are strongly associated with cancer progression and poor patient survival [6,7]. In addition to signals from B7s, signals from molecules of the T-cell immunoglobulin and mucin-domain (TIM) family also have very important immunological functions [8]. Three TIM-containing molecules (TIMs), TIM-1, TIM-3, and TIM-4, have been identified in humans [8]. In addition to playing an essential role in the regulation of immune responses, TIMs have been found to actively participate in tumorigenesis [9]. However, whether these proteins participate in the pathogenesis of parapharyngeal liposarcoma has not yet been reported.

## Case presentation

**Figure 1:**
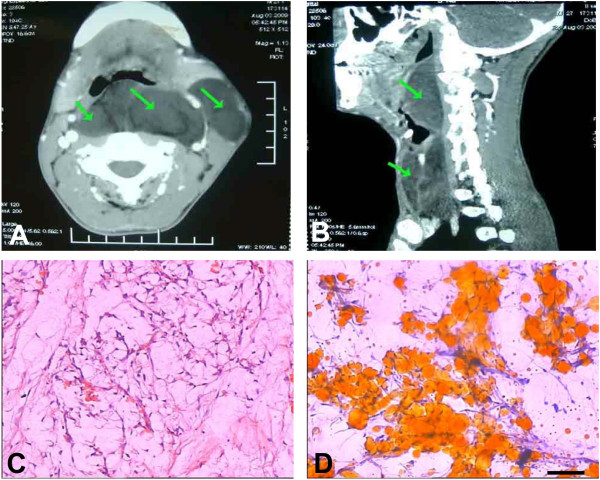
**The histopathological and pathological characteristics of the patient’s parapharyngeal liposarcoma, as analyzed by CT examination, H&E staining and Sultan-III staining.** (**A**) An axial image from the CT scan shows a large parapharyngeal mass of fat density. (**B**) A sagittal image from the CT scan shows a soft tissue mass at the midline. (**C**) Photomicrographs of the liposarcoma. H&E staining; scale bar = 20 μm. (**D**) The high level of lipid deposition in this parapharyngeal liposarcoma was visualized by sultan-III staining. The arrow indicates the mass, and the scale bar = 20 μm.

**Figure 2:**
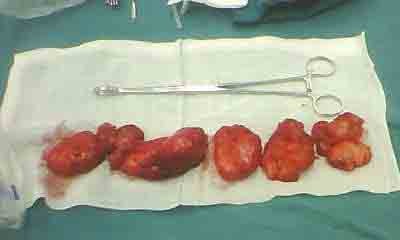
Several homogenous, yellow-tan masses were found during surgical removal.

A 30-year-old male patient who presented with a pharyngeal cavity mass, which had been present for 2 years, was referred to our department. The clinical syndrome included obstructive sleep apnea symptoms (i.e., respiratory disturbances, excessive daytime somnolence, and morning headache) and difficulty swallowing. The radiological examination (CT) revealed a low-density irregular solid lesion on the posterior wall of the oropharynx and laryngopharynx, which descended to the superior mediastinum and extended to the left parapharyngeal space and sternocleidomastoid muscle. The boundaries of the lesion were clear, and the lesion’s density was nonuniform. Several septations inside the lesion were observed. The CT values of the lesion at the epiglottis and the vocal folds were 11 HU and minus 30 HU, respectively. After enhanced scanning, there was no apparent enhancement of the lesion: the surrounding tissue and blood vessels were squeezed and shifted, but the neighboring sclerotin of the cervical vertebrae was not invaded [Figure 1A and B]. The mass was removed *via* a transcervical approach; several homogenous, yellow-tan masses were found, the biggest of which was 7×7×6 cm [Figure 2]. The patient remained tumor-free at 6 months and is now tolerating a regular diet, postoperatively.

### Immunohistochemical staining and results

Fresh or formalin-fixed paraffin-embedded samples were sliced at 3 μm thick, and the sections were prepared for H&E staining, Sultan-III staining and immunohistochemical detection. H&E staining confirmed that the mass exhibited strong malignant changes [Figure 1C]. In addition, a high level of lipid deposition in the sections was observed by Sultan-III staining [Figure 1D].

The sections were pressure cooked for 15 min in 10 mM citrate buffer (pH 6.0) for antigen retrieval, after being dewaxed with xylene and rehydrated using a graded series of ethanol. The sections were then incubated in phosphate-buffered saline (PBS) containing horse serum albumin, and the primary antibodies were added for the reaction, which was performed overnight at 4°C. Then, the second antibody was added, and the sections were incubated at 37°C for 30 min. Finally, the sections were treated with 3% H_2_O_2_, to reduce endogenous peroxidase activity, and the reaction was visualized with DAB.

**Figure 3:**
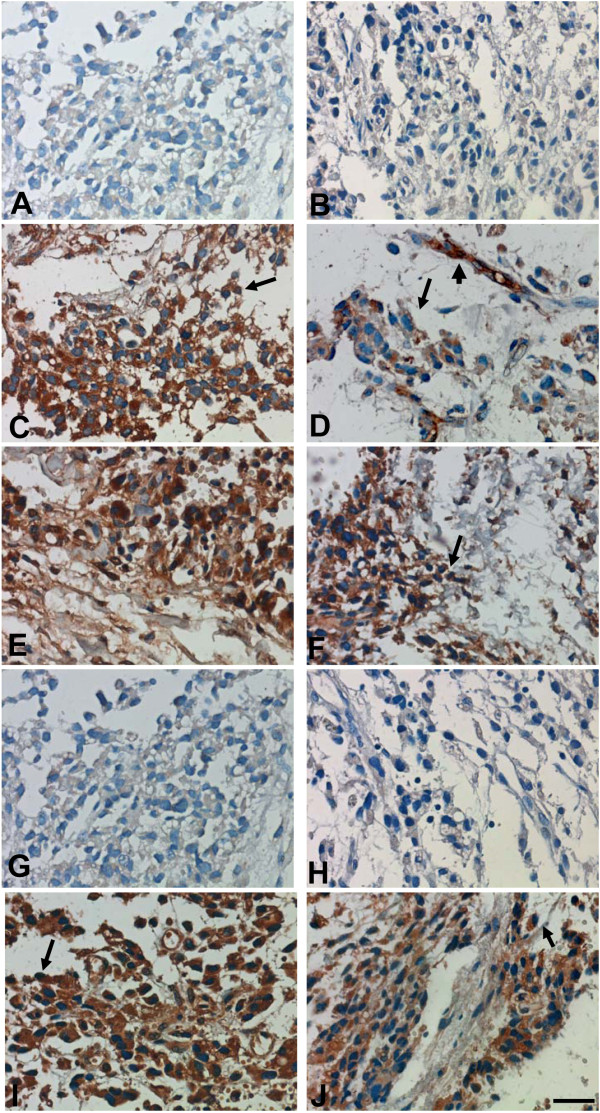
**The expression of B7s and TIM-containing molecules in parapharyngeal liposarcoma sample sections, as detected by immunohistochemistry.** (**A**) Mouse IgG isotype control antibodies showed no positive staining. (**B**) Goat IgG isotype control antibodies showed no positive staining. (**C**) B7-H1 was expressed on liposarcoma cell aggregates. (**D**) B7-DC was expressed on endothelial cells (capillaries). (**E**) B7-DC was expressed on liposarcoma cell aggregates. (**F**) B7-H3 was expressed on liposarcoma cell aggregates. (**G**) B7-H4 was absent from liposarcoma cell aggregates. (**H**) TIM-1 was absent from liposarcoma cell aggregates. (**I**) TIM-3 was expressed on liposarcoma cell aggregates. (**J**) TIM-4 was expressed on liposarcoma cell aggregates. The arrows indicate carcinoma cells, and the arrowheads indicate capillaries. Scale bar = 20 μm.

Immunohistochemistry showed that B7-H1-, B7-DC- and B7-H3-positive cells were present in the sample and were distributed throughout the tissue sections. Specifically, these B7 superfamily molecules were found on cell membranes and in the cytoplasm. In addition to being expressed on liposarcoma cells, B7-DC was also found on the capillaries, whereas B7-H4 was absent from the whole tissue section [Figure 3C-G]. The immunohistochemical analysis also showed that TIM-3- and TIM-4-positive but not TIM-1-positive liposarcoma cells were present. Similar to the characteristic expression pattern of B7s, TIM-3 and TIM-4 were found on cell membranes and in the cytoplasm, and their respective positive cells were distributed throughout the tissue sections [Figure 3H-J].

## Discussion

Parapharyngeal liposarcomas are extremely rare, and only a few cases have been described in the literature. They are very slow-growing tumors and do not cause symptoms until they reach a large size [1,3]. The most common symptoms include an abnormal sensation in the throat, noisy breathing, hoarseness, dyspnea, dysphagia and obstructive sleep apnea [1-3]. Diagnosing these tumors is difficult. CT and/or MRI evaluation of the head and neck is very important, although histological confirmation is critical [1]. Here, we present the case of a 30-year-old male patient who complained of obstructive sleep apnea symptoms and difficulty swallowing for 2 years. The combination of radiological examination (CT) and immunohistochemical analysis, using Sultan-III staining and H&E staining methods, demonstrated that the lesion was a typical parapharyngeal liposarcoma.

The mechanisms responsible for liposarcoma development are very complicated and are not fully understood. Expression of co-stimulatory B7-related molecules by cancer tissues has been reported. B7-related molecules can provide positive or negative signals to local T cells and regulate cancer development. The selective enhancement of T cell activation, using the CTLA-4-Ig protein or PD-1 blocking antibodies, has been demonstrated to be a suitable strategy for cancer immunotherapy [10]. The TIM-containing molecules, including TIM-1, TIM-3 and TIM-4, are newly discovered proteins that are actively involved in tumor development. The expression of TIM-3 has been found on tumor cells, including those from non-small cell lung cancers (NSCLCs), hepatitis B virus-associated hepatocellular carcinoma (HBV-HCC), renal cell carcinoma (RCC) and follicular B cell non-Hodgkin’s lymphoma [11]. The level of TIM-3 was found to closely correlate with both tumor dissemination and prognosis [12]. Nevertheless, the expression and anatomic distribution of TIMs in liposarcoma has not been reported. In this study, an immunohistochemical assay showed that the liposarcoma cells expressed some members of the B7 superfamily, including B7-H1, B7-DC and B7-H3. Moreover, the expression of TIM-containing molecules, such as TIM-3 and TIM-4, was also observed.

## Conclusion

We presented a case of parapharyngeal liposarcoma, which revealed the presence of carcinoma-associated B7 and TIM-containing molecules within the tumor tissue. Taken altogether, the data further indicated that these proteins likely actively participate in the pathogenesis of this disease.

## Consent

Written informed consent was obtained from the patient for publication of this Case Report and any accompanying images. A copy of the written consent is available for review by the Editor-in-Chief of this journal.

## Competing interests

None of the authors have any conflicts of interest related to this manuscript.

## Authors’ contributions

HL participated in the discussion for histological diagnosis and manuscript preparation. XZ collected the clinical data and postoperative clinical follow-up of the patient. QR was responsible for CT detection and LW is preparing the manuscript. All authors read and approved the final manuscript.
